# Effects of Pre-cooling and Cooling Breaks on Thermoregulatory, Physiological and Match Running Responses During Football in Moderate and Hot Temperatures

**DOI:** 10.1007/s40279-025-02325-z

**Published:** 2025-11-15

**Authors:** Edgar Schwarz, Catarina B. Oliveira, Monica Duarte Muñoz, Agustín Alanis, Marcela Alanis, Aldo Lara, Alfredo Freeze, Júlio A. Costa, Tim Meyer, Rob Duffield

**Affiliations:** 1 Institute of Sports and Preventive Medicine, Saarland University, Saarbrücken, Saarland Germany; 2 School of Sport, Exercise & Rehabilitation, Faculty of Health,, University of Technology Sydney, Ultimo, NSW Australia; 3 NOVA Medical School, Faculdade de Ciências Médicas, NMS, FCM, Universidade NOVA de Lisboa, Lisbon, Portugal; 4 FPF Academy, Portuguese Football Federation, FPF, Oeiras, Portugal; 5 Club Tigres UANL, San Nicolas de los Garza, Nuevo León Mexico

## Abstract

**Purpose:**

This study investigated the effects of pre-cooling and cooling breaks on thermoregulatory, hydration and running responses in football (soccer) players in moderate and hot temperatures.

**Methods:**

Forty male youth footballers participated in at least two of four matches, during which core body temperature (*T*_core_), heart rate (HR), match running, hydration and perceptual responses were measured. Cooling breaks (CBs), consisting of ice-cold towels and drinks, were compared to drinking breaks (DBs), consisting of passive rest and a temperate drink, applied at the same timeframes. Both were used as pre-cooling for 10 min before the warm-up, before the pre-match, during half-time and during additional 3-min cooling breaks at the 25th minute of each half. Initially, 20 players performed two crossover matches in 25 °C wet-bulb globe temperature (WBGT) receiving cooling (CB_25_) and drinking (DB_25_). A second group of 20 players played a regular match in 25 °C WBGT with no breaks (NB_25_) and then a match in 33 °C WBGT during which they received either cooling (CB_33_) or drinking breaks (DB_33_).

**Results:**

In CB_25_, players felt cooler (*p* < 0.001) and less fatigued (*p* < 0.045) than in DB_25_, without differences in match running (*p* > 0.20), HR_mean_ (*p* > 0.35) or *T*_core_ (*p* > 0.09). Players in CB_25_ sweated less (*p* = 0.005) and drank less (*p* = 0.002), resulting in no significant difference in body mass loss compared to DB_25_. In CB_33_, players had lower HR_mean_ (*p* = 0.007), similar total distance (*p* = 0.21), lower peak *T*_core_ (*p* < 0.001) and lower body mass loss (*p* = 0.007) compared to NB_25_. In DB_33_, players reduced moderate (12–18 km/h; *p* = 0.007) and high-speed running distance (18–24 km/h; p = 0.002) but had similar peak *T*_core_ (*p* = 0.71) and body mass loss (*p* = 0.95) to that in NB_25_.

**Conclusions:**

In general, high *T*_core_ values and body mass losses were observed even when playing in moderate heat. Both drinking and cooling breaks attenuated the continuous *T*_core_ rise, but using cooling also improved player perceptions in moderate temperatures. In hotter temperatures, cooling breaks further lowered *T*_core_ and body mass loss compared to using only drinking breaks.

**Trial Regsistry:**

German Clinical Trials Register: DRKS00032208.

**Supplementary Information:**

The online version contains supplementary material available at 10.1007/s40279-025-02325-z.

## Key Points

**Table:** 

Pre-cooling and additional 3-min breaks per half help mitigate the continuous core body temperature rises in football, even when playing in moderate heat.
Incorporating cooling strategies during breaks reduces perceptual fatigue and thermal sensation in moderate temperatures and further mitigates *T*_core_ rise and dehydration in severe heat.
Sweat loss and dehydration were high but introducing additional breaks facilitates greater fluid intake while adding cooling strategies reduces sweat loss.

## Introduction

High temperatures are a concern for football (soccer) organizations due to their potential implications for player health and performance [1–5]. In such temperatures, footballers typically reduce total and high-speed running [4, 6, 7], likely to mitigate heat strain based on exertion levels [8]. Despite this, the physical demands of football remain high under hot conditions, leading to substantial rises in core body temperature (*T*_core_). Peak *T*_core_ values of 39.6 ± 0.3 °C and 39.7 ± 0.1 °C have been reported during matches at 36 °C and 43 °C, respectively, with some players exceeding 40 °C [6, 9]. While athletes may tolerate hyperthermia without visible impairments, such high *T*_core_ values are concerning due to the increased risk of exertional heat illnesses [10]. These concerns can range from mild symptoms, such as headaches, cramps, and nausea, to severe exertional heat strokes [11], which is one of the leading causes of exercise-related death among athletes [12].

The best strategies to mitigate excessive heat strain in sports, such as scheduling competitions in cooler conditions (i.e., late evenings) or acclimatizing athletes to heat [12], are often not feasible for football. In professional football, the scheduling of matches is often determined by television contractual obligations, and the congested season schedules rarely allow time to heat acclimatize for the required durations (> 10 days), though short-term heat acclimation or post-exercise heating has been proposed as a possible solution [13–15]. In amateur settings, rescheduling matches is restricted by the availability of playing grounds, and acclimatization may be challenging due to limited financial resources and time constraints of amateur players. Therefore, acute interventions like hydration and cooling strategies or additional breaks per half are proposed [1, 2]. Football federations have introduced drinking breaks (DBs) or cooling breaks (CBs) when temperature or wet-bulb globe temperature (WBGT) exceeds specific thresholds. For example, the Fédération Internationale de Football Association (FIFA) mandates 3-min breaks when WBGT exceeds 32 °C [16], while other organizations recommend breaks at 26 °C WBGT [17]. In laboratory-based football simulations, additional 3-min breaks per half reduced *T*_core_ by 0.25 °C (using cold drinks) and 0.28 °C (using cold drinks and ice towels) in 35 °C (30 °C WBGT), with no differences between these interventions [18]. However, in 40 °C (32 °C WBGT), *T*_core_ reductions were greater when using cold drinks and ice towels during breaks (− 0.39 °C) compared to using breaks without drinks or cooling (− 0.28 °C) [19]. This aligns with evidence that cooling interventions become increasingly effective as heat strain rises [20]. Thus, cooling breaks may well be an effective heat-mitigating strategy in football.

In laboratory-based football simulations, pre-match and half-time cooling reduced *T*_core_ by 0.2–0.3 °C [21–23] or 0.6–0.9 °C [24–26], depending on the cooling dose and duration. Reduced thermal sensation [24, 25, 27] and improved endurance performance [24–26, 28] were also reported. However, methods like 60-min pre-cooling [21, 24], full-body cold-water immersions [29], frequent fluid intake [24], or wearing ice vests during play [23] are impractical for real matches, due to the time and logistical constraints of match days and the continuous and dynamic nature of the game. Even ice-filled towels [18, 19], common in individual sports [30], are logistically challenging for team sports. Although larger cooling doses yield stronger effects [31, 32], research outcomes need to meet the logistical constraints of football matches. Furthermore, studies omit warm-ups [18, 21, 22, 24], which are standard match-day elements alongside travel, changing and waiting periods, which are also not incorporated in laboratory studies, thus reducing the transferability of the findings. Finally, heat strain in these laboratory studies remained lower compared to field settings [6, 9], where external motivation (e.g., chasing the ball, scoring) may drive players to sustain high intensities despite the heat. Therefore, field-based observations are needed, replicating match physical demands and the feasibility and applicability of cooling interventions [33].

Only one study has investigated pre-cooling in real football matches in 26 °C WBGT [34]. It reported that 20 min of pre-cooling initially reduced *T*_core_ by a large effect; however, the effects disappeared after the warm-up. Although no significant effects for improved physical performance were found, moderate effects indicated more total distance was covered when cooling was applied. Nonetheless, reductions in sweat loss, perceived exertion and thermal strain were noted [34]. This highlights the challenges of transferring laboratory findings to field settings and underscores the need for repeated cooling throughout the match, though further field-based research is necessary.

This study investigated the effect of pre-cooling and cooling breaks on thermoregulatory, hydration and running responses in footballers in moderate and hot temperatures. The cooling strategy consisting of ice-cold towels and drinks applied pre-match, half-time and during additional 3-min cooling breaks (CBs) was compared to performing passive rest and a temperate drink during drinking breaks (DBs) in matches held at 25 °C WBGT and 33 °C WBGT. It was hypothesized that both CB and DB would mitigate the continuous rise in *T*_core_, but that CBs would reduce the heat strain more, while increasing match running, hydration status and perceptual markers compared to DBs.

## Methods

### Participants and Study Overview

**Fig. 1:**
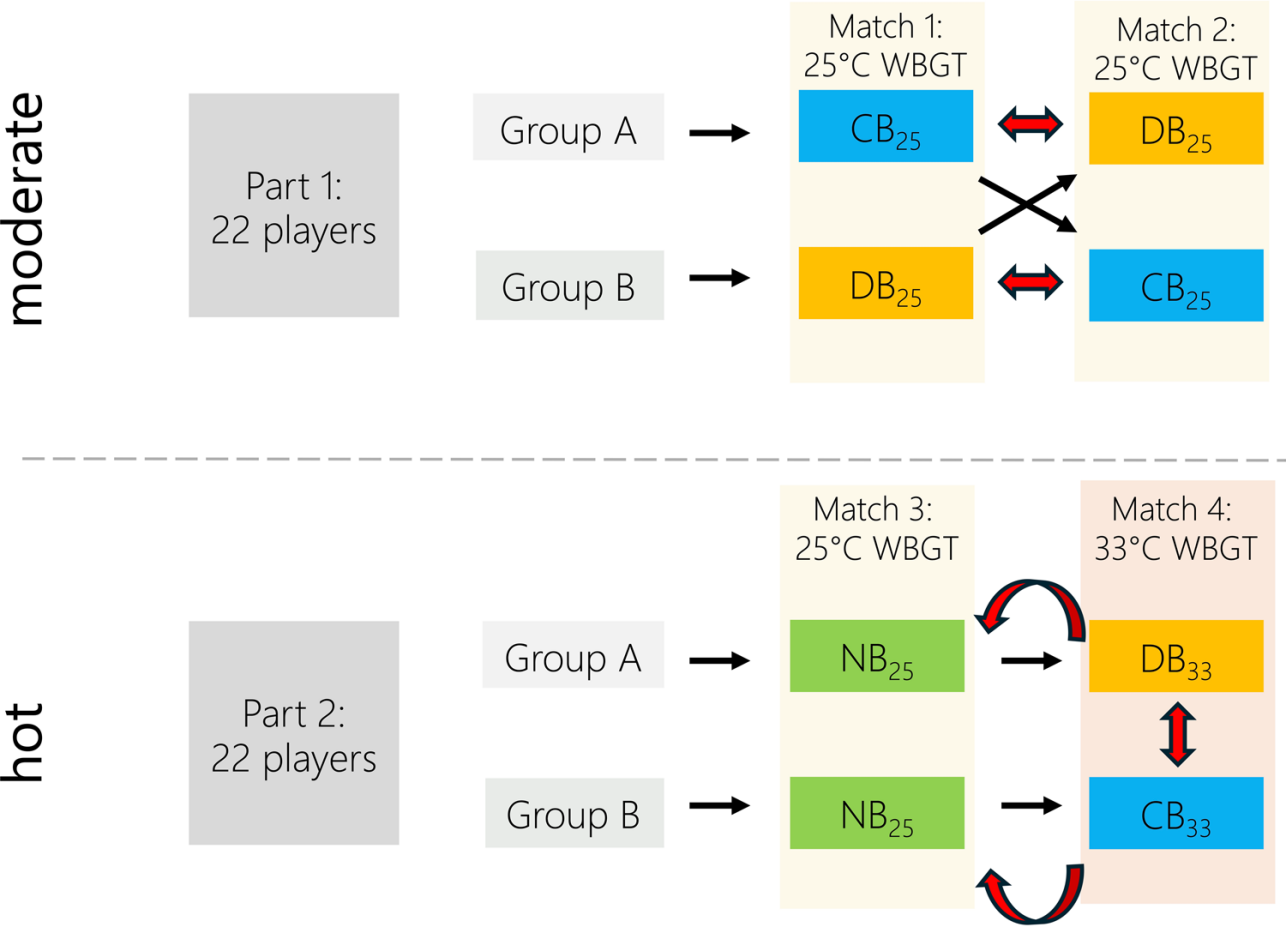
Visualization of overall design with two distinct data collections in moderate and hot conditions (the red arrows indicate which groups were compared using linear mixed models). *WBGT* wet bulb globe temperature, *CB*_*25*_ cooling breaks in 25 °C WBGT, *DB*_*25*_ drinking breaks in 25 °C WBGT, *NB*_*25*_ no breaks in 25 °C WBGT, *CB*_*33*_ cooling breaks in 33 °C WBGT, *DB*_*33*_ drinking breaks in 33 °C WBGT

Forty highly trained (Tier 3; [35]) male footballers from a professional Mexican club’s youth academy were recruited to participate in this study (goalkeepers excluded). The participants were between 16 and 19 years old, with a weight of 69 ± 6 kg, height of 175 ± 8 cm and body mass index (BMI) of 23 ± 2. Thermoregulatory responses to exercising under heat stress have been shown to be similar in children compared to adults [36]. Female participants were not included in this study, as it was only possible to organise four male-based games for the present study. Future replications of this research involving female athletes are warranted, as outcomes might differ [37]. All participants were involved in three to four training sessions and one to two matches per week. In Monterrey (Mexico), the average daily temperature peaks are 32 °C in May, 35 °C in July, and 32 °C in September. The matches for this study were held in October 2023 (Part 1) and June 2024 (Part 2), thus all participants were seasonally acclimatized, having trained in hot conditions for over 2 weeks prior to testing. Each player participated in at least one of two distinct data-collections (Parts 1 and 2; presented in Fig. 1).

In Part 1, 22 football players (aged 17 ± 1 years), played two matches 3 days apart in an average of 25 °C WBGT (Match 1: 24 °C WBGT; Match 2: 27 °C WBGT) receiving cooling (CB_25_) or drinking breaks (DB_25_) in a crossover design. Players were split into pairs of two by the teams’ coaches, based on age, body mass, playing position and skill, to then create two teams of similar strength. Participants were then randomized via an online randomiser by one of the researchers into treatment groups, with the condition to distribute treatments equally across teams and positions (i.e., each team had half of the players in CB_25_ during the first match, and CB_25_ and DB_25_ had an equal number of defenders, midfielders and attackers). As the goalkeepers were excluded for the analysis and there were two cases of minor injuries, which led to study discontinuation, 18 participants remained for the analysis. Substitutions continued the match for the injured players but were not included in the data collection. For Part 2, a second group of 22 football players (aged 18 ± 1 years; four overlapping with the first group) was planned to follow the same crossover protocol in hotter conditions and were randomized to groups as described in Part 1. However, due to cooler temperatures than forecasted, Match 3 was held in 25 °C WBGT, the decision was taken to play this as a regular match with no pre-cooling and no cooling breaks (NB_25_). Match 4 was held in 33 °C WBGT, and the initial randomization was used to distribute participants into receiving cooling (CB_33_) or drinking breaks (DB_33_). Again, goalkeepers were excluded from the analysis leading to a total of 20 participants included in the analysis. Overall, this resulted in 76 individual outfield player observations across four matches. However, for some measurements up to 19 observations had to be excluded retrospectively due to technical issues, such as the telemetric pills remaining in the stomach (*n* = 15) or being excreted too early (*n* = 4), global positioning system (GPS) device failures (*n* = 1), or participant injury (*n* = 2). The number of valid observations for each measurement is shown in Table 1, alongside the group and environmental conditions. During an initial familiarization session, all study procedures and measurements were explained via a presentation of pictures and infographics. Following this familiarization session, participants provided written informed consent to participate in the study. The study was pre-registered at the German Clinical Trials Register (DRKS-ID: DRKS00032208) and institutional ethics approval was granted by the Ethics Committee of the Faculty for Human and Business Sciences of Saarland University (No: 23-14).

**Table 1:** Environmental conditions and the number of players with valid data per condition (missing data were due to injuries, GPS and heart rate (HR) device failures and/or core body temperature (*T*_core_) pills remaining in the stomach)

Condition	Match	WBGT	T	RH	*n*—Total	*n*—GPS	*n*—HR	*n*—*T*_core_
DB_25_	Match 1 & 2	25.5 °C	28.9 °C	40.7%	18	18	16	11
	Match 1 Match 2	26.9 °C 24.0 °C	29.3 °C 28.4 °C	50.6% 30.8%				
CB_25_	Match 1 & 2	25.5 °C	28.9 °C	40.7%	18	17	15	13
	Match 1 Match 2	26.9 °C 24.0 °C	29.3 °C 28.4 °C	50.6% 30.8%				
NB_25_	Match 3	25.5 °C	26.0 °C	80.9%	20	19	19	19
DB_33_	Match 4	33.0 °C	36.5 °C	42.1%	10	10	10	10
CB_33_	Match 4	33.0 °C	36.5 °C	42.1%	10	10	10	9

*WBGT* wet-bulb globe temperature*, T* temperature, *RH* relative humidity, *n-total.* number of overall players per conditions, excluding goalkeepers and injury dropout, *n—GPS* number of players with valid running data, *n—HR* number of players with valid heart rate data, *n—T*_*core*_ number of players with valid core temperature data, *CB*_*25*_ cooling breaks in 25 °C WBGT, *DB*_*25*_ drinking breaks in 25 °C WBGT, *NB*_*25*_ no breaks in 25 °C WBGT, *CB*_*33*_ cooling breaks in 33 °C WBGT, *DB*_*33*_ drinking breaks in 33 °C WBGT

### Time Schedule and Logistics

**Fig. 2:**
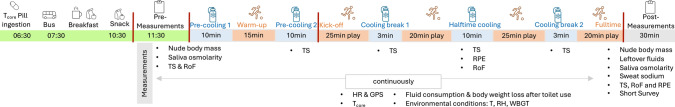
Timetable of Match 4, exemplary of an experimental match day. *HT* half-time, *HR* heart rate, *GPS* global positioning system, *TS* thermal sensation, *RoF* rating of fatigue, *RPE* rating of perceived exertion

For Part 1 (CB_25_ and DB_25_), participants met at 7:30 a.m. and started a 1-h bus ride to the match venue. Then they received a standardized breakfast (8:30 a.m.), rested and later consumed another standardized snack (11:30 a.m.). Pre-match measurements began at 12:30 p.m. and were followed by the first 10-min pre-cooling in CB or passive rest in DB (1:15 p.m.), a 15-min warm-up, the second 10-min pre-cooling in CB or passive rest in DB (1:45 p.m.), and the match onset (2:00 p.m.). Matches consisted of two 45-min halves, separated by a 15-min half-time (including 10 min of cooling for CB or drinking for DB), with each half containing an additional 3-min break per half at the 25th and 70th minute where players received CB or DB, except during the NB_25_ condition, where no additional breaks were performed. Post-match measurements started immediately after the match, including nude body mass measurements, downloading *T*_core_ data, hydration measurements and the collection of the sweat patches. All measurements were completed within 30 min post-match. For Part 2 (NB_25_, DB_33_ and CB_33_), the protocol (presented in Fig. 2) remained the same but began 2 h earlier, with the initial meeting at 5:30 a.m. and kick-off at 12:00 p.m., due to logistical requirements.

During CBs, participants received large cold towels (70 × 130 cm) covering the head, neck, shoulders and upper back. These towels, kept in 5–7 °C iced water, were re-dunked in the water after 5 min during each 10-min break and were only minimally wrung out before they were applied, to maximize the coldness of the intervention. Participants were also provided with two individual bottles: one with 500 ml of water and another with 500 ml of a commercial sports drink (Powerade, The Coca-Cola Company, Atlanta, GA, USA), both at 5 °C. Participants were instructed to finish the sports drink by the second CB (70th min) to standardize carbohydrate intake, while water could be consumed and refilled ad libitum. During DB, participants performed a passive rest and were given the same fluids at 17 °C. To blind participants to the intervention, the sports drink was mixed with a flavouring and colouring agent (Bebida Frutal, Nature’s Factory, Santa Catarina, Mexico) and described as a “sports drink” to improve performance in the heat. The researchers performing the data collection were not blinded to the intervention. In the NB_25_ condition, the match started after the warm-up and no mid-half breaks were conducted. Participants received the same drinks at 17 °C and were instructed to finish the sports drink by half-time.

### Measurements

#### Match Running and Heart Rate

All participants were fitted with a GPS device (WIMU Pro Elite Tracking System, Hudl, Lincoln, NE, USA), validated to measure running in team sports [38] and a heart rate (HR) monitor (Garmin HRM Dual™, Garmin International, Inc., Olathe, KS, USA). Running outcomes were recorded at 100 Hz and reported over the full match and per quarter (Q1: kick-off—pre first cooling break; Q2: post first cooling break—half-time; Q3: half-time—pre second cooling break; Q4: post second cooling break—full-time). Metrics are presented as “per minute”, to reflect time-relative match running and include total distance (TD), moderate-speed running distance (MSRD: 12–18 km/h), high-speed running distance (HSRD: 18–24 km/h) and sprinting distance (SD: > 24 km/h). HR is presented as peak and mean values for the full match and per playing quarter.

#### Core Body Temperature

*T*_core_ was measured continuously every 30 s using telemetric pills (eCelcius Performance, BodyCap, Hérouville-Saint-Clair, France), validated for continuous *T*_core_ monitoring [39]. Participants ingested the pills at 7:30 or 8:30 a.m. (Part 1) or 5:30 a.m. (Part 2), allowing at least 4 h to pass into the intestines. Participants with unphysiological *T*_core_ values (< 35 °C) after drinking, indicating the pill remaining in the stomach, were excluded. *T*_core_ is presented as overall match peak and peak values at key time points (baseline, post pre-cooling 1, post warm-up, post pre-cooling 2, post play 1 (25 min), post additional break 1, post play 2 (45 min), post half-time break, post play 3 (70 min), post additional break 2, post play 4 (90 min)).

#### Hydration and Fluid Balance

Participant’s fluid balance was assessed by measuring nude body mass pre- and post-match, as well as pre- and post-bathroom use (to monitor toilet breaks). After baseline measurements, fluid intake was monitored using individualized bottles, with participants instructed not to spit, spill or shower with water from them and measuring remaining fluid in the bottle post-match. Sweat loss (not corrected for gas exchange) was then calculated accordingly [40]:

$${\text{Sweat Loss }} = {\text{ Weight}}_{{{\mathrm{Baseline}}}} - {\text{ Weight}}_{{{\mathrm{Post}}}} + {\text{ Fluid Intake }} - {\text{ Urine Loss}}$$

Saliva osmolarity (SOMS) was measured pre- and post-match using a mobile device (MX3 LAB Pro, MX3 Diagnostics, Austin, TX, USA), which has been shown to be reliable to assess changes in hydration status [41]. Participants refrained from consuming food or liquid for 15 min prior to the measurement, then swallowed all their saliva, before presenting a freshly produced saliva sample on the tip of their tongue for measurement. Participants were categorized as hydrated (< 65 mOsm), mildly dehydrated (65–100 mOsm), moderately dehydrated (100–150 mOsm) and severely dehydrated (> 150 mOsm). These categories are based on SOSM values reported in literature and distributions across the MX3 costumer population. Participants started with no significant differences in pre-match saliva osmolality (DB_25_: 61.6 ± 20.1 mOsm; CB_25_: 62.1 ± 14.1 mOsm; p = 0.89; DB_33_: 63.5 ± 18.4 mOsm; CB_33_: 69.1 ± 16.9 mOsm; NB_25_: 69.2 ± 28.0 mOsm; all *p* ≥ 0.49).

#### Perceptual Measures

Rating of fatigue (RoF) [42], rating of perceived exertion (RPE) [43], and thermal sensation (TS) [44] were recorded (Fig. 2). All scales were translated into Spanish, introduced at the familiarization session, and shown to the participants each time they were assessed. Following the match, after players had showered and eaten a post-match meal, they completed a survey on their perceptions of the interventions. This was a shortened and adapted version of an intervention implementation survey [45]. Participants were asked to rate how much they liked each intervention on a scale from − 5 (did not like it at all) to + 5 (liked it very much) and whether they perceived any performance benefits from − 5 (did not perceive performance benefits at all) to + 5 (perceived a lot of performance benefits).

### Statistical Analyses

A sample size calculation was performed based on the study of Brown et al. (2024). The Cohen f effect size for difference in final *T*_core_ was 0.32 (DB vs. CB) and 0.64 (CB vs. NB). Based on these effect sizes, an alpha of 0.05 and power of 0.80, the optimal sample size needed for a cross-over study would be between 11 and 39 per group, to investigate the effect of cooling breaks on *T*_core_. The initially planned sample size of 20 participants per group was determined by logistical factors (i.e., number of outfield players in a football match) and was further reduced due to unpredicted environmental conditions and technical issues (outlined earlier), which is a limitation of this study. Values are reported as means and standard deviations. Due to small and dependent samples, linear mixed models were performed to test group differences, accounting for repeated measures and variability across subjects. Thus, to investigate differences between CB_25_ and DB_25_, linear mixed models with a random effect per participant were used. In total, 32 models were built, one for each outcome measure (running and HR: 5; *T*_core_: 13; hydration: 4; perceptions: 10) to investigate differences between conditions in the first sample. CB_33_ and DB_33_ were compared directly per linear mixed model with a random effect per team. Further, participants in CB_33_ and DB_33_ were also compared to their individual “reference” in NB_25_, using linear mixed models with a random effect per player. Therefore, three models, one comparing independent samples (CB_33_–DB_33_) and two comparing dependent samples (CB_33_–NB_25_, DB_33_–NB_25_) were built for each of the 32 outcome measures. This design is outlined in Fig. 1, with red arrows indicating the groups that were compared with linear mixed models. Model outcomes are reported as estimates and 95% confidence intervals (CIs), standardized estimates (*β*), and 95% CIs and explained variance (*R*^2^). Effects were categorized as small (*β* > 0.1), medium (*β* > 0.3) or large (β > 0.5; [46]) and explained variance was categorized as small (*R*^2^ > 0.01), medium (*R*^2^ > 0.09) or large (*R*^2^ > 0.25; [47]). Significance was set at *α* = 0.05. All analysis and figures were conducted using R (Version 4.4.1) with packages lme4, pwr, jtools, dplyr, ggplot2 and reshape2.

## Results

### Drinking (DB_25_) and Cooling Breaks (CB_25_) in 25 °C Wet-Bulb Globe Temperature (WBGT)

**Fig. 3:**
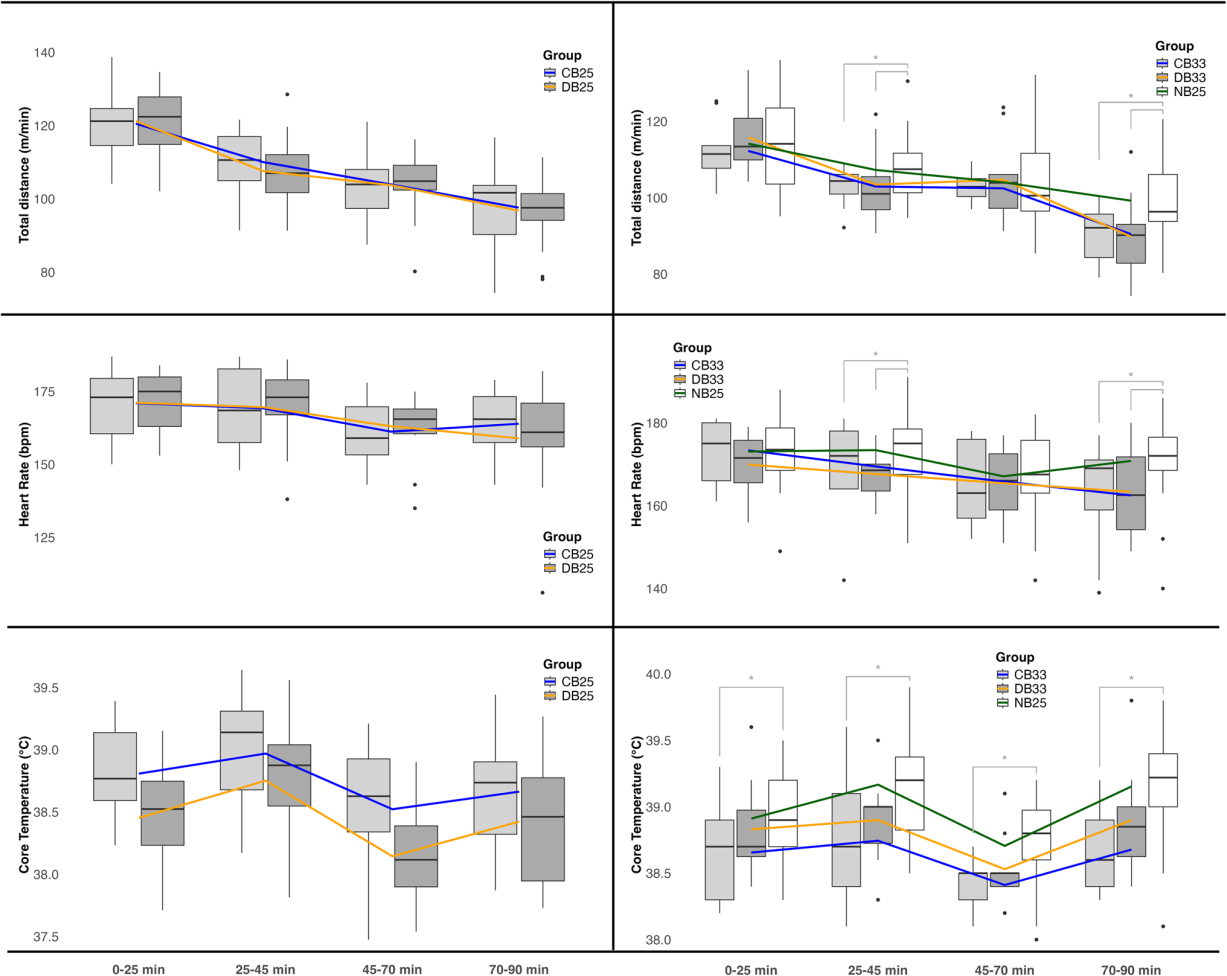
Mean total distance covered, heart rate and *T*_core_ (core body temperature) per playing quarter in each condition (centre line = median; box = interquartile range (IQR); whiskers = smallest and largest values within 1.5 times the IQR; individual points = outliers; *Significant (*p* < 0.05) difference between those two groups). *WBGT* wet bulb globe temperature, *CB*_*25*_ cooling breaks in 25 °C WBGT, *DB*_*25*_ drinking breaks in 25 °C WBGT, *NB*_*25*_ no breaks in 25 °C WBGT; *CB*_*33*_ cooling breaks in 33 °C WBGT, *DB*_*33*_ drinking breaks in 33 °C WBGT

Total distance covered was 108.0 ± 8.1 m/min in DB_25_ and 108.5 ± 8.0 m/min in CB_25_ (*p* = 0.61), with no differences observed per playing quarter (all *p* ≥ 0.25; Fig. 3) or at different speed zones (all *p* ≥ 0.73; Table 2).

**Table 2:** Running performance in Part 1 comparing cooling (CB_25_) compared to drinking breaks (DB_25_) in 25 °C wet-bulb globe temperature (WBGT) and Part 2 comparing cooling (CB_33_) and drinking breaks (DB_33_) in 33 °C WBGT to no breaks (NB_25_) in 25 °C WBGT

	Mean (SD)				Estimate (95% CI)	*β* (95% CI)	*R*^2^ marg	*p*-value
Part 1	CB_***25***_		DB_***25***_					
Total (m/min)	108.5 (8.0)		108.0 (8.1)	CB_25_–DB_25_	− 0.5 (− 2.3, 1.4)	− 0.1 (− 0.5, 0.3)	0.00	0.61
Moderate speed (m/min)	25.6 (5.5)		25.5 (6.4)	CB_25_–DB_25_	− 0.1 (− 1.7, 1.5)	− 0.0 (− 0.4, 0.4)	0.00	0.90
High speed (m/min)	7.9 (2.8)		7.7 (2.5)	CB_25_–DB_25_	− 0.2 (− 1.3, 0.9)	− 0.1 (− 0.4, 0.3)	0.00	0.73
Sprint (m/min)	1.6 (1.2)		1.6 (1.0)	CB_25_–DB_25_	− 0.0 (− 0.5, 0.5)	0.0 (− 0.4, 0.4)	0.00	0.98

Part 2	CB_***33***_	DB_***33***_	NB_***25***_					
Total (m/min)	102.7 (4.5)	104.3 (9.9)	106.5 (9.9)	CB_33_–DB_33_	1.6 (− 5.5, 8.7)	0.1 (− 0.4, 0.6)	0.01	0.66
				DB_33_–NB_25_	2.7 (− 0.6, 5.9)	0.4 (− 0.1, 0.8)	0.01	0.13
				CB_33_–NB_25_	2.7 (− 1.2, 6.8)	0.3 (− 0.3, 0.9)	0.05	0.21
Moderate speed (m/min)	20.4 (2.5)	20.3 (5.1)	23.1 (5.8)	CB_33_–DB_33_	− 0.1 (− 3.7, 3.6)	− 0.0 (− 0.5, 0.5)	0.00	0.96
				DB_33_–NB_25_	3.5 (0.8, 6.1)	0.5 (0.1, 0.9)	0.08	0.007*
				CB_33_–NB_25_	1.8 (− 0.6, 4.3)	0.4 (− 0.2, 0.9)	0.07	0.16
High speed (m/min)	5.5 (1.64)	6.0 (1.93)	6.8 (2.19)	CB_33_–DB_33_	0.5 (− 1.1, 2.1)	0.1 (− 0.4, 0.7)	0.02	0.57
				DB_33_–NB_25_	1.3 (0.3, 2.3)	0.5 (0.1, 0.9)	0.09	0.02*
				CB_33_–NB_25_	0.7 (− 0.9, 2.3)	0.2 (− 0.3, 0.8)	0.04	0.38
Sprint (m/min)	1.2 (0.5)	1.2 (0.8)	1.1 (0.9)	CB_33_–DB_33_	0.0 (− 0.6, 0.6)	0.0 (− 0.5, 0.5)	0.00	0.94
				DB_33_–NB_25_	− 0.1 (− 0.4, 0.2)	− 0.2 (− 0.7, 0.4)	0.00	0.52
				CB_33_–NB_25_	− 0.2 (− 1.0, 0.6)	− 0.1 (− 0.7, 0.4)	0.01	0.67

*SD* standard deviation, *CI* confidence interval, *β* standardized estimate, *R*^*2*^* marg.* explained variance of the fixed effects, *m/min* meters per minute, *Total* total distance covered, *Running* distance covered 12–18 km/h, *High-speed running* distance covered 18.1–24 km/h, *Sprint* distance covered above 24 km/h

Mean HR during the match was 166 ± 11 beats/min (bpm) in DB_25_ and 166 ± 11 bpm in CB_25_, with no differences overall (*p* = 0.78) or per playing quarter (all *p* ≥ 0.35; Fig. 3).

**Fig. 4:**
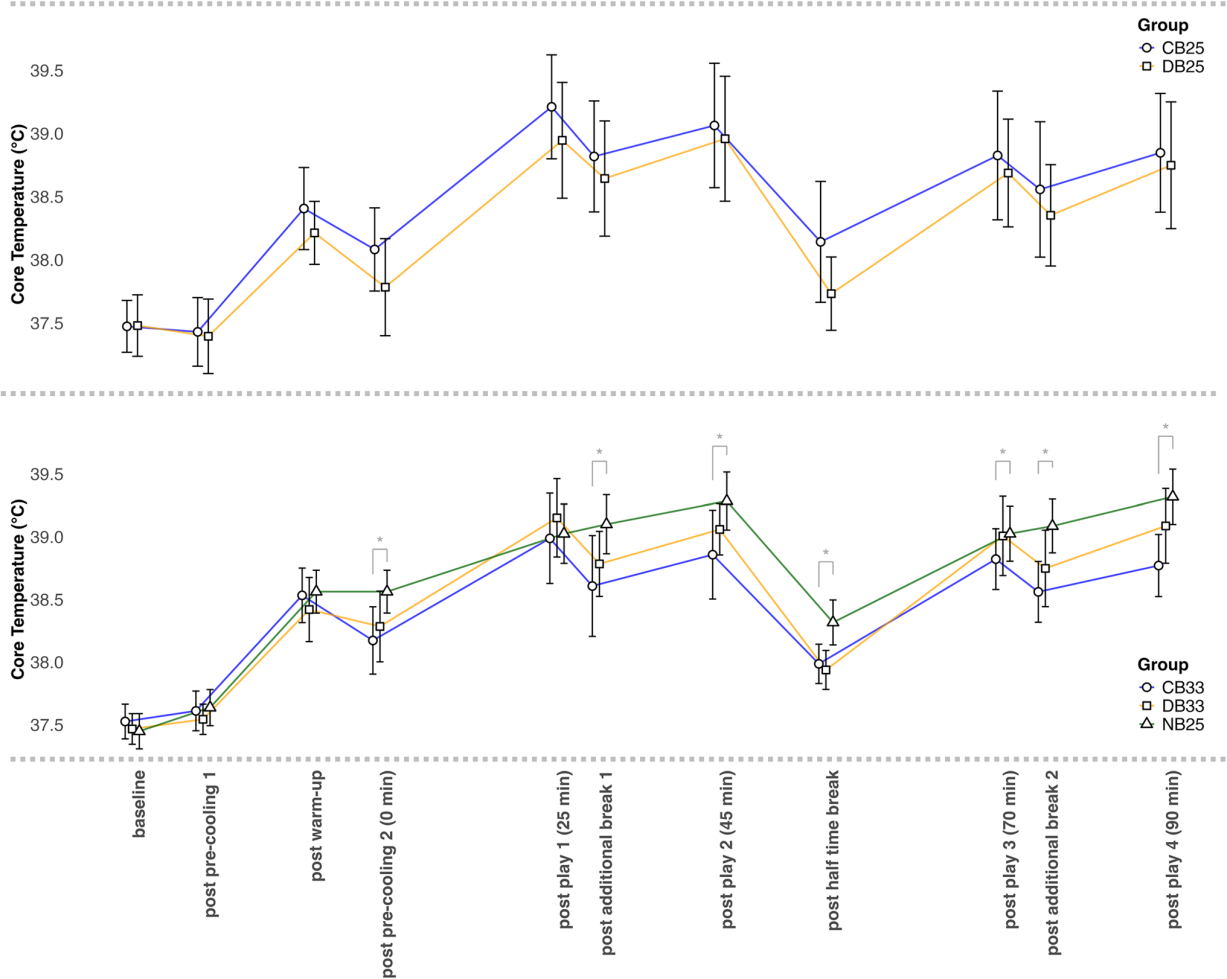
Core body temperature (*T*_core_) peaks at specific time-points throughout the match day for each condition (circle, square, triangle = group mean; whiskers = 95% confidence intervals; lines = connecting the means representing the mean change over that period; *significant (*p* < 0.05) difference; all significant differences were players in CB_33_ remaining at lower *T*_core_ to what these players had achieved in NB_25_). *WBGT* wet bulb globe temperature, *CB*_*25*_ cooling breaks in 25 °C WBGT, *DB*_*25*_ drinking breaks in 25 °C WBGT, *NB*_*25*_ no breaks in 25 °C WBGT, *CB*_*33*_ cooling breaks in 33 °C WBGT, *DB*_*33*_ drinking breaks in 33 °C WBGT

*T*_core_ did not significantly differ between conditions at any time-point (all *p* ≥ 0.09; Fig. 4). Resting *T*_core_ prior to the match was 37.5 ± 0.3 °C in DB_25_ and 37.5 ± 0.3 °C in CB_25_ (*p* = 0.96). Mean *T*_core_ during the match was 38.4 ± 0.5 °C in DB_25_ and 38.7 ± 0.5 °C in CB_25_ (*p* = 0.28), with no significant differences overall or per playing quarter (all *p* ≥ 0.15; Fig. 3). Peak *T*_core_ was 39.1 ± 0.6 °C in DB_25_ and 39.3 ± 0.5 °C in CB_25_ (*p* = 0.42). The mean *T*_core_ reduction during the additional breaks per half was − 0.32 ± 0.2 °C in DB_25_ and − 0.37 ± 0.2 °C in CB_25_ (*p* = 0.27).

Post-match saliva osmolarity increased to 89.7 ± 49.0 mOsm in DB_25_ and 78.7 ± 29.7 mOsm in CB_25_, but was not different between the groups (*p* = 0.43). Sweat loss was 0.38 L (0.11, 0.66) higher in DB_25_ (*β* = 0.41; *R*^2^ = 0.13; *p* = 0.005) and fluid intake was 0.19 L (0.07, 0.32) higher in DB_25_ (*β* = 0.43; *R*^2^ = 0.10; *p* = 0.002) compared to CB_25_. This resulted in a similar body mass loss (*p* = 0.69) (Table 3).

**Table 3:** Hydration markers in Part 1 comparing cooling (CB_25_) with drinking breaks (DB_25_) in 25 °C wet bulb globe temperature (WBGT) and Part 2 comparing cooling (CB_33_) and drinking breaks (DB_33_) in 33 °C WBGT with no breaks (NB_25_) in 25 °C WBGT

	Mean (SD)				Estimate (95% CI)	*β* (95% CI)	*R*^2^ marg.	*p*-value
Part 1	CB_***25***_		DB_***25***_					
Sweat loss (L)	2.7 (0.4)		3.0 (0.6)	CB_25_–DB_25_	0.4 (0.1, 0.7)	0.4 (0.1, 0.7)	0.13	0.005*
Fluid intake (L)	1.2 (0.3)		1.4 (0.3)	CB_25_–DB_25_	0.2 (0.1, 0.37)	0.4 (0.1, 0.7)	0.10	0.0018*
Δ body mass (%)	− 2.1 (0.5)		− 2.2 (1.2)	CB_25_–DB_25_	0.1 (− 0.4, 0.6)	0.1 (− 0.3, 0.4)	0.00	0.69
Δ SOSM (mOsm)	20.4 (33.4)		33.2 (49.5)	CB_25_–DB_25_	11.6 (− 16.7, 39.8)	0.1 (− 0.2, 0.5)	0.02	0.43

Part 2	CB_***33***_	DB_***33***_	NB_***25***_					
Sweat loss (L)	3.0 (0.7)	3.7 (0.6)	2.7 (0.7)	CB_33_–DB_33_	0.7 (0.2, 1.2)	0.5 (0.1, 1.0)	0.19	0.006*
				DB_33_–NB_25_	− 0.9 (− 1.5, − 0.4)	− 0.6 (− 1.0, − 0.2)	0.34	< 0.001*
				CB_33_–NB_25_	− 0.4 (− 0.8, − 0.1)	− 0.5 (− 0.9, − 0.0)	0.09	0.02*
Fluid intake (L)	− 1.7 (0.3)	− 2.1 (0.7)	− 1.1 (0.3)	CB_33_–DB_33_	0.4 (− 0.0, 0.9)	0.4 (− 0.1, 0.9)	0.15	0.06
				DB_33_–NB_25_	− 1.0 (− 1.4, − 0.6)	− 0.7 (− 1.1, − 0.4)	0.51	< 0.001*
				CB_33_–NB_25_	− 0.7 (− 0.9, − 0.5)	− 0.8 (− 1.1, − 0.5)	0.62	< 0.001*
Δ body mass (%)	− 2.0 (0.8)	− 2.4 (1.1)	− 2.5 (0.8)	CB_33_–DB_33_	0.4 (− 0.4, 1.1)	0.2 (− 0.3, 0.7)	0.04	0.32
				DB_33_–NB_25_	− 0.0 (− 0.9, 0.9)	− 0.0 (− 0.5, 0.5)	0.00	0.95
				CB_33_–NB_25_	0.5 (0.1, 0.9)	0.5 (0.1, 0.9)	0.12	0.007*
Δ SOSM (mOsm)	48.6 (45.3)	65.8 (29.6)	28.6 (28.8)	CB_33_–DB_33_	17.2 (− 16.2, 50.6)	0.2 (− 0.3, 0.7)	0.05	0.33
				DB_33_–NB_25_	− 25.7 (− 47.2, − 5.4)	− 0.5 (− 0.9, − 0.1)	0.17	0.01*
				CB_33_–NB_25_	− 29.9 (− 63.6, 3.6)	− 0.4 (− 0.9, 0.1)	0.14	0.11

*SD* standard deviation, *CI* confidence interval, *β* standardized estimate, *R*^*2*^* marg.* explained variance of the fixed effects, *l* liter, Δ change, *SOSM* saliva osmolarity, *mOsm* milliosmole

In CB_25_, participants reported significantly lower RoF (*p* = 0.03), RPE (*p* = 0.01) and TS (*p* = 0.001) compared to DB_25_ (Table 4). The post-match survey showed participants liked and perceived performance benefits from both but favoured (median: + 3 vs. + 2; *β* = 0.35; *R*^2^ = 0.12; *p* = 0.02) and perceived greater performance benefits (median: + 3 vs. + 2; *β* = 0.41; *R*^2^ = 0.17; *p* = 0.01) from CB_25_.

**Table 4:** Perceptual markers in Part 1 comparing cooling (CB_25_) compared to drinking breaks (DB_25_) in 25 °C wet bulb globe temperature (WBGT) and Part 2 comparing cooling (CB_33_) and drinking breaks (DB_33_) in 33 °C WBGT to no breaks (NB_25_) in 25 °C WBGT

	Mean (SD)				Estimate (95% CI)	*β* (95% CI)	*R*^2^ marg.	*p*-value
Part 1	CB_***25***_		DB_***25***_					
RoF half-time	3.8 (1.2)		4.7 (1.5)	CB_25_–DB_25_	0.9 (0.1, 1.8)	0.3 (0.0, 0.7)	0.11	0.029*
RoF full-time	5.8 (1.3)		6.3 (1.3)	CB_25_–DB_25_	0.5 (0.1, 0.9)	0.4 (0.0 − 0.7)	0.04	0.045*
RPE half-time 1	3.8 (1.2)		4.7 (1.4)	CB_25_–DB_25_	0.8 (0.2, 1.5)	0.4 (0.1, 0.7)	0.09	0.006*
RPE half-time 2	5.4 (1.4)		5.9 (1.3)	CB_25_–DB_25_	0.4 (− 0.2, 1.1)	0.2 (− 0.1, 0.6)	0.03	0.19
TS pre-match	0.2 (1.7)		0.2 (1.7)	CB_25_–DB_25_	0.0 (0.0, 0.0)	0.0 (− 0.6, 0.6)	0.00	1.0
TS kick-off	− 1.7 (1.0)		0.9 (1.4)	CB_25_–DB_25_	2.7 (1.9, 3.5)	0.8 (0.5, 1.0)	0.50	< 0.001*
TS half-time	0.0 (1.4)		2.3 (1.3)	CB_25_–DB_25_	2.3 (1.5, 3.1)	0.7 (0.4, 0.9)	0.43	< 0.001*
TS full-time	1.3 (1.6)		1.9 (1.4)	CB_25_–DB_25_	0.7 (− 0.2, 1.5)	0.3 (− 0.1, 0.6)	0.05	0.13

Part 2	CB_***33***_	DB_***33***_	NB_***25***_					
RoF half-time	6.1 (0.3)	5.9 (0.9)	4.6 (1.4)	CB_33_–DB_33_	− 0.2 (− 0.8, 0.4)	− 0.2 (− 0.7, 0.3)	0.02	0.51
				DB_33_–NB_25_	− 1.3 (− 2.2, − 0.4)	− 0.5 (− 0.9, − 0.1)	0.18	0.002*
				CB_33_–NB_25_	− 1.6 (− 2.2, − 1.0)	− 0.8 (− 1.1, − 0.4)	0.56	< 0.001*
RoF full-time	8.0 (1.3)	8.7 (0.7)	7.9 (1.1)	CB_33_–DB_33_	0.7 (− 0.2, 1.6)	0.4 (− 0.1, 0.8)	0.11	0.14
				DB_33_–NB_25_	− 1.1 (− 1.8, − 0.5)	− 0.6 (− 0.9, − 0.2)	0.24	< 0.001*
				CB_33_–NB_25_	0.2 (− 0.2, 0.6)	0.2 (− 0.3, 0.8)	0.01	0.34
RPE half-time 1	5.4 (1.1)	5.3 (1.6)	4.2 (1.6)	CB_33_–DB_33_	− 0.1 (− 1.3, 1.1)	− 0.0 (− 0.5, 0.5)	0.00	0.87
				DB_33_–NB_25_	− 1.1 (− 2.7, 0.5)	− 0.3 (− 0.8, 0.2)	0.08	0.21
				CB_33_–NB_25_	− 1.2 (− 1.9, − 0.5)	− 0.5 (− 0.9, − 0.2)	0.25	< 0.001*
RPE half-time 2	8.1 (1.7)	7.8 (1.1)	7.5 (1.7)	CB_33_–DB_33_	− 0.3 (− 1.6, 1.0)	− 0.1 (− 0.6, 0.4)	0.01	0.65
				DB_33_–NB_25_	− 0.5 (− 1.9, 0.9)	− 0.2 (− 0.7, 0.4)	0.03	0.49
				CB_33_–NB_25_	− 0.4 (− 1.2, 0.4)	− 0.2 (− 0.8, 0.3)	0.02	0.34
TS pre-match	2.7 (1.2)	2.6 (0.7)	1.5 (1.2)	CB_33_–DB_33_	− 0.1 (− 0.9, 0.7)	− 0.1 (− 0.6, 0.4)	0.00	0.82
				DB_33_–NB_25_	− 0.8 (− 1.5, − 1.1)	− 0.5 (− 0.9, 0.0)	0.20	0.014*
				CB_33_–NB_25_	− 1.6 (− 2.2, − 1.1)	− 0.7 (− 0.9, − 0.4)	0.31	< 0.001*
TS kick-off	2.6 (1.1)	3.2 (0.4)	1.8 (0.9)	CB_33_–DB_33_	0.6 (− 0.1, 1.3)	0.4 (− 0.1, 0.8)	0.12	0.12
				DB_33_–NB_25_	− 1.8 (− 2.3, − 1.3)	− 0.9 (− 1.1, − 0.6)	0.72	< 0.001*
				CB_33_–NB_25_	− 0.5 (− 1.4, 0.4)	− 0.3 (− 0.7, 0.2)	0.06	0.27
TS half-time	3.3 (0.7)	3.8 (0.4)	1.2 (1.7)	CB_33_–DB_33_	0.5 (0.0, 1.0)	0.4 (− 0.0, 0.9)	0.17	0.04*
				DB_33_–NB_25_	− 2.4 (− 3.1, − 1.7)	− 0.8 (− 1.0, − 0.5)	0.60	< 0.001*
				CB_33_–NB_25_	− 2.4 (− 3.7, − 1.1)	− 0.6 (− 1.0, − 0.3)	0.40	< 0.001*
TS full-time	3.0 (0.8)	3.9 (0.3)	2.0 (1.8)	CB_33_–DB_33_	0.9 (0.4, 1.4)	0.6 (0.2, 1.0)	0.36	0.0012*
				DB_33_–NB_25_	− 1.8 (− 2.8, − 0.8)	− 0.6 (− 1.0, 0.2)	0.39	< 0.001*
				CB_33_–NB_25_	− 1.2 (− 2.3, − 0.2)	− 0.5 (− 0.9, − 0.0)	0.14	0.02*

*SD* standard deviation, *CI* confidence interval, *β* standardized estimate, *R*^*2*^* marg.* explained variance of the fixed effects, *RoF* rating of fatigue, *RPE* rating of perceived exertion, *TS* thermal sensation

### Drinking (DB_33_) and Cooling Breaks (CB_33_) in 33 °C WBGT

Total distance was significantly lower in quarters 2 and 4 for both DB_33_ and CB_33_ compared to NB_25_ (all *p* ≤ 0.022; Fig. 3), with no significant differences between DB33 and CB33 (all *p* ≥ 0.40). However, MSRD and HSRD were reduced in DB_33_, but not in CB_33_ compared to NB_25_ (Table 2).

Mean HR was 167 ± 8 bpm in DB_33_, 168 ± 8 bpm in CB_33_, and 171 ± 10 bpm in NB_25_. For participants in CB_33_ this was significantly lower compared to their values in NB_25_ (*β* = 0.53; *R*^2^ = 0.04; *p* = 0.007), but no significant difference was observed in DB_33_ (*β* = 0.16; *R*^2^ = 0.01; *p* = 0.51) (Fig. 3).

Resting *T*_core_ was similar across all conditions (DB_33_: 37.5 ± 0.2 °C; CB_33_: 37.5 ± 0.2 °C; NB_25_: 37.4 ± 0.3 °C; all p ≥ 0.25). Mean *T*_core_ in CB_33_ was 38.6 ± 0.3 °C, which was significantly lower than in NB_25_ (− 0.31 (0.14, 0.49) °C; *β* = 0.59, *R*^2^ = 0.16; *p* < 0.001). The participants in DB_33_ recorded a mean *T*_core_ of 38.7 ± 0.4 °C, which was not significantly lower compared to their mean *T*_core_ in NB_25_ (− 0.14 (− 0.16, 0.43) °C; *β* = 0.22, *R*^2^ = 0.04; *p* = 0.36). Peak *T*_core_ in CB_33_ was 39.1 ± 0.5 °C, significantly lower by 0.30 °C (0.14, 0.47) compared to the 39.4 ± 0.4 °C in NB_25_ (*β* = 0.60, *R*^2^ = 0.12; *p* < 0.001). In DB_33_, peak *T*_core_ was 39.3 ± 0.3 °C, not different to NB_25_ (*β* = 0.09, *R*^2^ = 0.00; *p* = 0.71). Direct comparison of DB_33_ and CB_33_ showed no significant difference in mean *T*_core_ (*p* = 0.33) or peak *T*_core_ (*p* = 0.23). Mean *T*_core_ per playing quarter is presented in Fig. 3 and *T*_core_ development throughout the match is visualized in Fig. 4. *T*_core_ was significantly lower in CB_33_ than NB_25_ from kick-off to full-time (all *p* < 0.001), except at the start of the first cooling break (*p* = 0.98). At full-time *T*_core_ was 0.62 °C (0.42, 0.83) lower in CB_33_ compared to what these participants reached in NB_25_ (*β* = 0.69; *R*^2^ = 0.48; *p* < 0.001) and 0.32 °C (− 0.02, 0.65) lower compared to participants in DB_33_, though this difference was not significant (*β* = 0.41; *R*^2^ = 0.16; *p* = 0.08). The mean *T*_core_ drop during the 3-min cooling and drinking breaks was similar between DB_33_ (0.31 ± 0.2 °C) and CB_33_ (0.32 ± 0.2 °C; *p* ≥ 0.76). However, during the second pre-cooling break, *T*_core_ dropped by − 0.4 ± 0.2 °C in CB_33_, which was 0.23 °C (0.00, 0.45) greater than the − 0.1 ± 0.3 °C drop in DB_33_ (*β* = − 0.43; *R*^2^ = 0.18; *p* = 0.049).

Post-match saliva osmolarity increased to 129.3 ± 39.7 mOsm in DB_33_ and 117.7 ± 50.3 in CB_33_, with a significantly higher increase in DB_33_ (*p* = 0.047) but not CB_33_ (*p* = 0.33) compared to NB_25_ (110.9 ± 30.5 mOsm). Sweat loss was higher in CB_33_ (*p* = 0.02) and DB_33_ (*p* < 0.001) compared to NB_25_, but fluid intake was also greater in CB_33_ (*p* < 0.001) and DB_33_ (*p* < 0.001; Table 3). This resulted in a significantly lower body mass loss in CB_33_ (*p* = 0.007) but not DB_33_ (*p* = 0.95) and a significantly bigger change in saliva osmolarity in DB_33_ (*p* = 0.01), but not CB_33_ (*p* = 0.11; Table 3).

Significant differences in RoF, RPE and TS are presented in Table 4. Direct comparison between DB_33_ and CB_33_ showed lower TS in CB_33_ from half-time to full-time (all *p* < 0.05). Participants rated liking both CB_33_ and DB_33_ highly (median: + 4) and perceived performance benefits from CB_33_ (median: + 3) and DB_33_ (median: + 2), with no significant differences between the conditions.

## Discussion

This study investigated the effects of cooling (CBs) and drinking breaks (DBs) on thermoregulatory, physiological and perceptual responses in 40 highly trained male youth footballers during four matches played at 25 °C and 33 °C WBGT. This field-based evidence shows how pre-cooling and additional breaks per half mitigate the continuous rise in *T*_core_, suggesting their utility for managing heat strain in footballers. Adding ice-cold drinks and towels during breaks reduced perceptual strain and sweat loss in milder conditions and further limited the *T*_core_ rise in hotter environments.

Match running did not differ between DB and CB in 25 °C WBGT. In 33 °C WBGT, total distance and mean HR were reduced in the second and fourth quarters for both DB_33_ and CB_33_ compared to NB_25_. This indicates participants adjusted workloads to manage heat strain, consistent with previous research [4, 7]. However, participants in DB_33_ reduced MSRD (12–18 km/h) and HSRD (18–24 km/h) compared to NB_25_, whereas players in CB_33_ maintained these, suggesting benefits from the cooling intervention in 33 °C WBGT. This may be linked to the lower *T*_core_, but also the lower thermal sensations observed in CB_33_, as previous research has shown that skin temperature and thermal perception can influence exercise intensity in the heat [48].

Despite a ~ 10.5 °C difference in ambient temperature and ~ 8 °C WBGT across matches, participants reached similar peak *T*_core_ values, with most (*n* = 43) exceeding 39.0 °C and some (*n* = 17) exceeding 39.5 °C. The highest mean *T*_core_ peak was observed in NB_25_, including one participant exceeding 40 °C, potentially due to the more continuous running in the match without breaks. This indicates tolerable but substantial heat strain even at 25 °C WBGT in a match without breaks for seasonally acclimatized participants. Similar peak *T*_core_ values in 25 and 33 °C WBGT may reflect the reduced running distances in 33 °C WBGT, underscoring the interplay between workload and thermal strain in football [49, 50]. This further enforces that heat strain, i.e., *T*_core_, and the risk of heat illnesses, are related to the exertion levels (i.e., running demands) and not environmental conditions alone [8, 49, 51].

The initial pre-cooling had no immediate effect on *T*_core_, but the second pre-cooling lowered *T*_core_ at kick-off by ~ 0.3 °C, suggesting that reducing warm-up time and allowing for a break before starting the match may be beneficial to increase heat storage capacity, as proposed in previous research [2, 12]. During the additional 3-min breaks, regardless of the environment and whether DB or CB was applied, mean *T*_core_ reduced by ~ 0.1 °C per minute, consistent with laboratory findings [18, 19]. However, overall heat strain in our study was higher and more comparable to field-based research [6, 9] than in laboratory conditions with a pre-break *T*_core_ of ~ 38 °C [18] or ~ 38.5 °C [19]. The continuous *T*_core_ rise observed in NB_25_ and previous field studies [6, 9] was attenuated when breaks were implemented, suggesting benefits even in milder heat (25 °C WBGT). FIFA’s cooling break is scheduled at the 30th minute of each half, which is based on research showing peak *T*_core_ occurs around this time [9, 18]. However, if peaks occur around the 30th minute, a break should precede this to prevent high *T*_core_ peaks. Therefore, in this study, breaks were held at the 25th minute. Since *T*_core_ did not exceed pre-break levels by the end of each half, this timing appears effective. The continuous mean *T*_core_ per group can be seen in the Online Supplementary Material, Fig. 1.

Adding ice-cold drinks (5 °C) and towels (5–7 °C) during breaks reduced perceptual strain and thermal sensation in milder heat and effectively lowered *T*_core_ in severe heat compared to drinking cool beverages (17 °C) alone. This aligns with research showing that cooling interventions are more effective in higher heat strain [52]. However, individual factors, such as fitness level, acclimatization and health status, co-determine heat strain; thus, cooling effectiveness might vary between players and across days [53]. Cooling strategies should therefore be tailored to individual player needs (and team resources) [12].

Sweat loss and body mass changes were high across all conditions, with 29 players losing over 3 L, five exceeding 4 L, 40 losing more than 2%, and ten more than 3% of body mass in one match. Existing literature shows that a dehydration > 2% impairs athletic performance, with endurance tasks being most affected [54]. Breaks provided hydration opportunities, facilitating greater fluid intake compared to the match without breaks. Cooling during breaks reduced sweat loss, potentially linked to a decreased thermoregulatory drive [55]. However, reduced sweating may have limited evaporative cooling, possibly explaining the lack of *T*_core_ differences between CB_25_ and DB_25_. In DB_25_, players matched higher sweat loss with increased fluid intake, maintaining body mass losses similar to CB_25_. This could suggest participants regulated fluid intake in response to the higher sweat loss. Although a similar outcome existed in 33 °C, participants in DB_33_ were not able to offset the higher sweat loss completely, leading to a higher body mass loss in DB_33_ compared to participants in CB_33_, potentially due to the sweat loss difference being too high to compensate with sufficient fluid intake. Notably, 64% of players began matches hypohydrated (saliva osmolarity > 65 mOsm). Although these categorizations are based on unpublished data and company thresholds (MX3), this is consistent with previous findings [56]. This is of particular concern, as hypohydration impairs the sweat response, reducing heat dissipation, thus increasing the heat strain [57]. This was confirmed in a study using football-simulating treadmill running in hot conditions, where higher HR and *T*_core_ were observed in hypohydrated compared to euhydrated participants [58]. Given the importance of hydration for performance, thermoregulation and preventing heat-related issues, educating players and stakeholders on proper hydration strategies seems essential [10–12].

Perceptual markers, including RoF and TS, were higher in 33 °C WBGT, confirming environmental stress as a key driver of perceived fatigue [59]. Cooling reduced the RoF and TS in CB_25_ versus DB_25_ and in CB_33_ versus DB_33_, demonstrating perceptual benefits even when *T*_core_ remained unchanged under lower heat stress. This is noteworthy, as interventions reducing thermal sensation, even if not affecting *T*_core_ or *T*_skin_, have been linked to improved performance [59]. In line with this, participants reported liking the cooling interventions highly and perceiving performance benefits in both 25 °C and 33 °C WBGT.

While this study provides novel outcomes in ecologically valid environments, several limitations should be noted. Missing *T*_core_ data, especially in the first sample (due to pills remaining in the stomach) reduced the initially planned sample size. The use of a different group in the second sample limited direct comparisons of DB_25_ and CB_25_ to NB_25_, DB_33_ and CB_33_. Unpredicted weather events led to a decision to adjust the study protocol in the third match. As a result, the two initially planned crossovers were not feasible and it decreased the sample size per group in the second part of the data collection. Additionally, the high relative humidity in NB_25_ should be considered, as WBGT is known to underestimate heat stress in humid conditions [60]. The participants were seasonally acclimatized by having exercised in high temperatures for 2 weeks prior to the study, as well as throughout their football careers in a country with high average temperatures. As acclimatization reduces heat strain, cooling interventions are expected to be more beneficial for non-acclimatized players [53]. Due to team availability, this study only included male youth players; however, it was shown that no relevant differences exist in the response to exercising under heat stress between children and adults, and heat policies may therefore be transferable [36]. Certainly, sex alone is not necessarily affecting the response to heat stress but differences between aerobic capacity and surface area-to-mass ratio between men and women might result in different physiological responses to heat stress [61, 62]. In football-simulating treadmill running, cooling breaks were less effective in females compared to men [19, 37]. Therefore, a replication with female players is needed. To summarize, small sample sizes, particularly in subgroup analyses, limit the generalizability of these findings. Future research should replicate this novel field-based approach with other cohorts and focus on (alternative) practical cooling strategies.

### Practical Implications

The findings suggest that pre-cooling strategies, such as an extended rest post warm-up, are simple and effective for reducing initial heat strain. Additional 3-min cooling or drinking breaks can mitigate the continuous rise in *T*_core_, and may prevent potentially dangerous *T*_core_ peaks even in moderate heat. The presented outcomes suggest an implementation of the breaks at the 25th minute, opposing recommendations of introducing breaks around the 30th minute. Incorporating cooling strategies during breaks may enhance player comfort in moderate ambient conditions and further reduce heat strain and sweat loss in severe heat. Drinking breaks facilitated greater fluid intake while cooling breaks reduced sweat loss. Hydration monitoring and education are essential, as players’ pre-match hydration status was often suboptimal, and sweat loss and dehydration during matches were high.

### Future Research

The findings of the study highlight the need for further applied investigations of match-day cooling strategies in football. This study should be replicated with other cohorts such as females, adults and unacclimatized players in both elite and amateur settings. Additionally, to identify optimal break structures, different cooling break and half-time durations should be examined. Furthermore, higher cooling doses could be tested, such as the use of an ice vest during warm-ups or more aggressive cooling during half-time, as these methods might be feasible in elite settings. However, it remains at least equally important to consider practical approaches for lower-competition settings, which may be more affected by high temperatures and have less medical support.

### Conclusions

In conclusion, pre-cooling and additional 3-min breaks per half, with or without cooling, mitigate the otherwise continuous *T*_core_ rise during football matches in moderate and high heat. Cooling reduced perceptual fatigue and thermal sensation in moderate temperatures and may further minimize the *T*_core_ rise and dehydration in hotter conditions. These findings support the implementation of additional 3-min breaks in football and highlight the potential benefits of cooling strategies.

## Supplementary Information

Below is the link to the electronic supplementary material.

Supplementary file1Supplementary Fig. 1: Continuous mean Tcore for each condition, with coloured highlights indicating when breaks took place. Abbreviations: CB_25 = cooling breaks in 25 °C WBGT; DB_25 = drinking breaks in 25 °C WBGT; NB_25 = no breaks in 25 °C WBGT; CB_33 = cooling breaks in 33 °C WBGT; DB_33 = drinking breaks in 33 °C WBGT) (JPG 972 KB)

## Declarations

### Data availability

The datasets generated and/or analyzed during the current study are not publicly available due to being team-specific data of Club Tigres UANL but are available from the corresponding author upon reasonable request.

### Clinical trial registration

This study was registered at the German Clinical Trials Register with the DRKS-ID: DRKS00032208, which can be accessed at: https://drks.de/search/en/trial/DRKS00032208.
