# Supplementary figures and images for: Effects of Pre-cooling and Cooling Breaks on Thermoregulatory, Physiological and Match Running Responses During Football in Moderate and Hot Temperatures
Source: Sports Med. 2025 Nov 15;56(2):573–87. doi: 10.1007/s40279-025-02325-z (PMC12982254; doi:10.1007/s40279-025-02325-z)

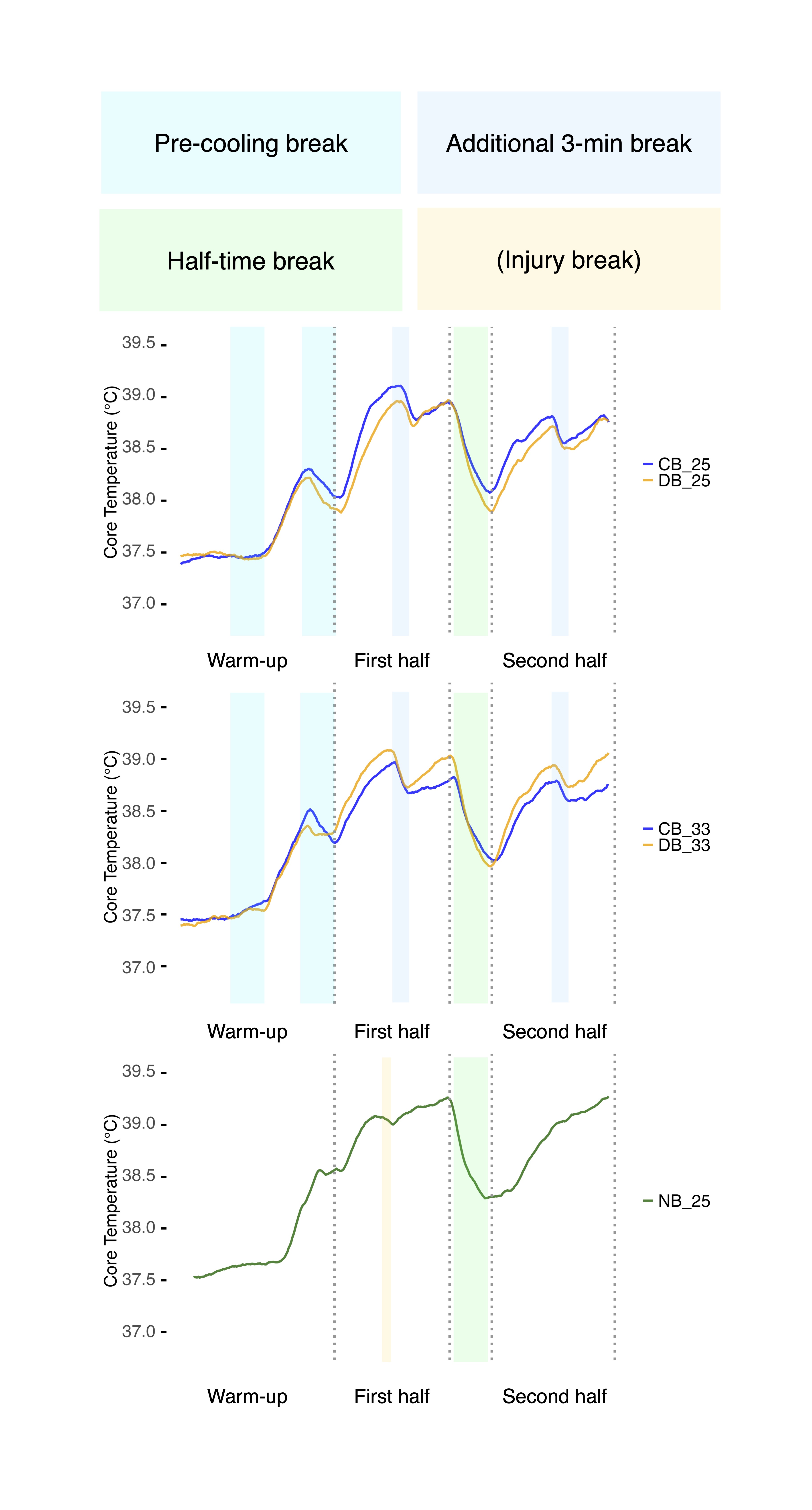

Supplement: Supplementary file 1 — Supplementary file1Supplementary Fig. 1: Continuous mean Tcore for each condition, with coloured highlights indicating when breaks took place. Abbreviations: CB_25 = cooling breaks in 25 °C WBGT; DB_25 = drinking breaks in 25 °C WBGT; NB_25 = no breaks in 25 °C WBGT; CB_33 = cooling breaks in 33 °C WBGT; DB_33 = drinking breaks in 33 °C WBGT) (JPG 972 KB) [file 40279_2025_2325_MOESM1_ESM.jpg]
